# A Digital Telehealth System to Compute Myasthenia Gravis Core Examination Metrics: Exploratory Cohort Study

**DOI:** 10.2196/43387

**Published:** 2023-04-19

**Authors:** Marc Garbey, Guillaume Joerger, Quentin Lesport, Helen Girma, Sienna McNett, Mohammad Abu-Rub, Henry Kaminski

**Affiliations:** 1 Department of Surgery School of Medicine & Health Sciences George Washington University Washington, DC United States; 2 ORintelligence LLC Houston, TX United States; 3 Laboratoire des Sciences de l'Ingénieur pour l'Environnement (LaSIE UMR-CNRS 7356) University of La Rochelle La Rochelle France; 4 Care Constitution Corporation Washington, DC United States; 5 Department of Neurology & Rehabilitation Medicine School of Medicine & Health Sciences George Washington University Washington, DC United States

**Keywords:** telehealth, telemedicine, myasthenia gravis, ptosis, diplopia, deep learning, computer vision, eyes tracking, neurological disease

## Abstract

**Background:**

Telemedicine practice for neurological diseases has grown significantly during the COVID-19 pandemic. Telemedicine offers an opportunity to assess digitalization of examinations and enhances access to modern computer vision and artificial intelligence processing to annotate and quantify examinations in a consistent and reproducible manner. The Myasthenia Gravis Core Examination (MG-CE) has been recommended for the telemedicine evaluation of patients with myasthenia gravis.

**Objective:**

We aimed to assess the ability to take accurate and robust measurements during the examination, which would allow improvement in workflow efficiency by making the data acquisition and analytics fully automatic and thereby limit the potential for observation bias.

**Methods:**

We used Zoom (Zoom Video Communications) videos of patients with myasthenia gravis undergoing the MG-CE. The core examination tests required 2 broad categories of processing. First, computer vision algorithms were used to analyze videos with a focus on eye or body motions. Second, for the assessment of examinations involving vocalization, a different category of signal processing methods was required. In this way, we provide an algorithm toolbox to assist clinicians with the MG-CE. We used a data set of 6 patients recorded during 2 sessions.

**Results:**

Digitalization and control of quality of the core examination are advantageous and let the medical examiner concentrate on the patient instead of managing the logistics of the test. This approach showed the possibility of standardized data acquisition during telehealth sessions and provided real-time feedback on the quality of the metrics the medical doctor is assessing. Overall, our new telehealth platform showed submillimeter accuracy for ptosis and eye motion. In addition, the method showed good results in monitoring muscle weakness, demonstrating that continuous analysis is likely superior to pre-exercise and postexercise subjective assessment.

**Conclusions:**

We demonstrated the ability to objectively quantitate the MG-CE. Our results indicate that the MG-CE should be revisited to consider some of the new metrics that our algorithm identified. We provide a proof of concept involving the MG-CE, but the method and tools developed can be applied to many neurological disorders and have great potential to improve clinical care.

## Introduction

With the COVID-19 pandemic, there was a rapid increase in the use of telemedicine in routine patient care [1] and in clinical trials that moved to video evaluations to maintain subject follow-up [2]. Telemedicine was already commonly used for acute stroke care and was in development for Parkinson disease, but the vast majority of neurologists were not using such approaches and were suddenly thrust into unfamiliar territory [3-5]. Diagnosis and monitoring of neuromuscular disorders, in particular, rely on a nuanced physical examination, and specialists would be particularly reticent to use telemedicine. However, telemedicine has great potential to provide improved assessment of aspects of neurological examinations, and facilitate patient monitoring and their education [6], while reducing patient burden in attending in-person clinic visits and potentially increasing access. Further, there is great potential for rigorous video assessment to enhance clinical trial performance, which could reduce the burden on study participants and thereby enhance recruitment and retention.

The Myasthenia Gravis Core Examination (MG-CE) [7] was recommended for telemedicine evaluation of patients with myasthenia gravis (MG), and it involves specific aspects of neurological examinations critical to the comprehensive assessment of patients with MG. The National Institutes of Health Rare Disease Clinical Research Network dedicated to MG, MGNet, initiated an evaluation to assess the feasibility and validity of MG-CE for use in future clinical trials. These assessments were video recorded using the software Zoom (Zoom Video Communications), and we used the evaluations performed at George Washington University with the following 2 objectives: (1) assess workflow efficiency by making the data acquisition and analytics fully automatic and (2) evaluate the potential to quantitate the evaluations.

## Methods

### MG-CE and Automatic Data Acquisition

The study used recorded telemedicine evaluations of individuals with a clinical- and laboratory-confirmed diagnosis of MG. The patients were provided instructions regarding their position in relation to the cameras and level of illumination, and were told to follow the examiner’s instructions. We used videos of 6 subjects recorded twice within 7 days to develop our algorithms. One normal control subject was used to evaluate the methodology prior to evaluating MG subject videos.

The MG-CE is summarized in Table 1, and a full description has been provided previously [7]. The examination required 2 broad categories of processing: (1) the computer vision algorithm to analyze video focusing on eye or body motions and (2) the analysis of the voice signal, which requires a completely different category of signal processing methods. We describe successively each of the techniques used in these categories and summarize the digitalization process in Table 2.

**Table 1:** Myasthenia Gravis Core Examination exercises and evaluation metrics [7].

Variable	Normal (0)	Mild (1)	Moderate (2)	Severe (3)
Eyelid droop (ptosis)	No ptosis	Eyelid above the pupil	Eyelid at the pupil	Eyelid below the pupil
Double vision (right/left)	No diplopia with a gaze of 61 seconds	Diplopia with a gaze of 11-60 seconds	Diplopia with a gaze of 1-10 seconds	Immediate diplopia
Cheek puff	Normal “seal”	Transverse pucker	Opposes lips but air escapes	Cannot perform the exercise
Tongue to cheek	Normal: full convex deformity in the cheek	Partial convex deformity in the cheek	Able to move the tongue to the cheek, but no deformity	Cannot perform the exercise
Counting to 50	No dysarthria at 50	Dysarthria at 30-49	Dysarthria at 10-29	Dysarthria at 1-9
Arm strength	No drift for >120 seconds	Drift at 90-119 seconds	Drift at 10-89 seconds	Drift at 0-9 seconds
Single-breath count	Count of ≥30	Count of 25-29	Count of 20-24	Count of <20
Sit-to-stand maneuver	No difficulty	Slow with effort but no hands	Need to use hands	Unable to stand unassisted

**Table 2:** Summary of our algorithm tool box to assist the clinician with the Myasthenia Gravis Core Examination.

Exercise	Description	Observation	Metric	Digital tool
Ptosis	Patients hold their gaze up for 60 seconds.	Weakness of the upper eyelid and eyelid going above the pupil.	Distance between the eyelid and the pupil, and distance between the upper and lower eyelids.	High-definition camera and eye segmentation.
Double vision	Patients hold their gaze right and then left for 60 seconds.	Misalignment of the eyes and moment of double vision.	Track the distance between anatomic landmarks such as the upper/lower lid, and pupil and iris left and right boundaries.	High-definition camera and eye segmentation.
Cheek puff	Patients puff their cheeks and hold it.	Assess muscle strength and fatiguability. Extent of puffiness at baseline and versus external pressure placed on the cheeks. Symmetry of cheek puff (left vs right).	Track face feature variation, mouth curvature, and dimension in particular.	Depth camera or Lidar. High-definition camera with face landmark monitoring. Track change of illumination in the region of interest.
Tongue pushing	Patients use their tongue to push the cheek.	Tongue muscle strength and symmetry.	Track face feature variation, mouth curvature, dimension, and orientation in particular.	Depth camera or Lidar. High-definition camera with face landmark monitoring. Track change of illumination in the region of interest.
Counting to 50	Patients count out loud from 1 to 50.	Assess for respiratory muscle fatigue and shortness of breath.	Loudness of the voice. Various types of spectral analysis of the voice and mouth motion. Energy metric of the voice.	Lip tracking and sound analysis of the exercise clip.
Arm strength	Patients hold their arms straight.	Assess for muscle fatigue via sustained abduction of the arm.	Track body pose and different angles. Length of time the patient can hold the arm in the pose. Trajectory of the arm over time.	Pose detection on high-definition images.
Single-breath test	Patients count with only 1 breath.	Assess for respiratory muscle fatigue.	Length of the breath.	Lip tracking and sound analysis of the exercise clip.
Sit-to-stand maneuver	Patients stand up with and without crossing their arms.	Assess for muscle fatigue. Ability of the patient to stand without using the arms for assistance.	Body pose tracking. Compare standing up speed between clips.	Pose detection on high-definition images.

### Deep Learning and Computer Vision Analysis

#### Machine Learning to Track Body Landmarks and Face Landmarks

**Figure 1:**
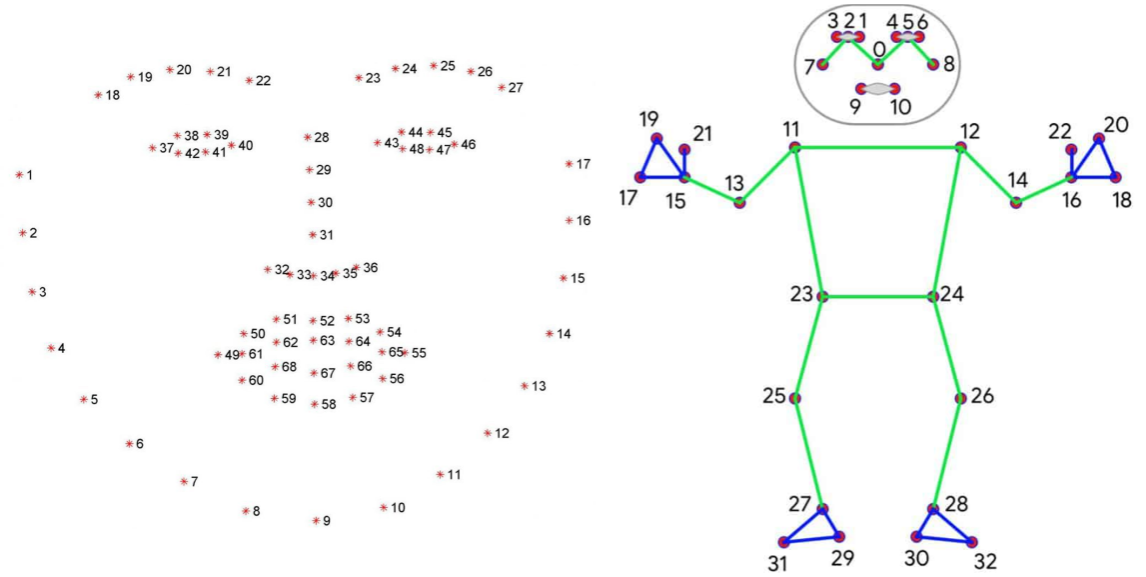
Pretrained machine learning models used with characteristic points.

Tracking faces or all body motions has become a standard tool [8] thanks to publicly available deep learning libraries with a standard model (Figure 1). To track body positions during the test of arm position fatigue and the sit-to-stand maneuver (Figure 1), we used a deep learning model that is publicly available (the pretrained machine learning model BlazePose GHUM 3D from MediaPipe) (Figure 1) [9]. For eye detection, we first needed to localize the face in the video frame.

Among the most commonly used algorithms [10,11], we chose OpenCV’s implementation of the Haar Cascade algorithm [12], based on the detector from Lienhart et al [13]. Our criteria to select the method were speed and reliability for real-time detection.

To focus on the regions of interest (ROIs) of the eyes and lids, we used the pretrained DLib 68 points facial landmark detector that is based on the shape regression approach [14,15]. It is a machine learning algorithm that places 68 characteristic points on a detected face. The model is pretrained on the I-BUG 300-W data set, which is comprised of 300 face pictures (Figure 1) [16]. This software was used for the assessment of ptosis and eye position, as well as for the test of counting to 50 and the single-breath test in order to document lip reading and tracking of jaw motion (Table 1).

Overall, both libraries provided robust results and could be used to annotate the video in real time for the ROIs. However, we found that the accuracy of the landmark points in the model of Figure 1 obtained by this library was not adequate to provide metrics that could be used in eye motion assessment in the context of a standard telehealth session. Therefore, we developed a hybrid method that began from deep learning to identify the ROIs and refine the search for the pupil, eyelid, and iris as described next.

#### Eye and Lid Image Segmentation

**Figure 2:**
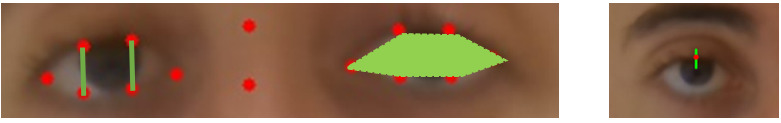
Approximations on ptosis to assess the field of view: distance between the upper and lower eye lids (left), eye area opening (center), and distance from the upper lid to the pupil (right).

Assessment of ptosis and ocular motility requires precise tracking of the eyelid, pupil, and iris. Precise metrics of these measures have been developed [17-20]. Established techniques to detect the iris location [21] are the circular Hough transform [22] and the Daughman algorithm method [23]. However, we found that these methods lack robustness due to their insensitivity to the low resolution of the ROIs of the eyes, poor control for illumination of the subject, and specific eye geometry consequent to ptosis. The eye image in a standard Zoom meeting may not be bigger than about 40 pixels wide and 20 pixels high. Liu et al [24] assessed eye movements for a computer-aided examination, but with highly controlled data and a highly controlled environment. We did not have optimum control of telehealth footage with patients at home, and the eye region has only one-tenth, at best, of the image frame dimension. Therefore, we took a more versatile approach that began with the ROI given by the previous deep learning library that we had used and then concentrated on a local search of the iris boundary, pupil center, and upper/lower eyelid (Figure 2). Since we started from a good estimate of the ROI for the eye, we used a combination of a local gradient method and clustering technique to compute the spatial coordinate and distance between landmarks of interest, and we have described this in the Results section. There are 2 classes of assessment depending on whether we compute the geometric dimension on an individual image or the dynamic of eye motion on video clips. We retrieved, for example, the relaxation time of the eyelid versus equilibrium, with some of the patients performing both eye exercises (Figure 2). However, there is no mention of such a metric in the core examination [11]. The incorporation of this new information in the standard core remains to be determined.

#### Body Image Segmentation

To have reproducible results with the entire view of the body during the examination, we tested our telehealth platform Inteleclinic on 1 patient and several control subjects. The pretrained machine learning model BlazePose GHUM 3D from MediaPipe [9] has been evaluated extensively, so we only provide some examples of the results obtained with the MG-CE. The arms of the patient are extended for 2 minutes during the exercise, and we used the segments joining the landmark point (12) to (14) to track the right arm position and the landmark point (11) to (13) to track the left arm position (Figure 1B). We computed the angle formed by the arm’s segment as described above and the horizontal line going through the landmark points (11) and (12) of the upper torso in the model (Figure 1B). If the arms stay horizontal, the 2 angles we track for the model (Figure 1B) should be approximately zero. As the arm strength of the patient may fatigue during the exercise, the arms fall from the horizontal position, and the angle would decrease and become negative. A similar approach was used for the sit-to-stand exercise by tracking the hip landmarks (23) and (24) of the body motion model (Figure 1B).

#### Cheek Deformation

The ROI for cheek deformation was the polygon delimited by points (3), (15), (13), and (5) of model 1 (Figure 1A) for the cheek puff exercise. We could restrict this ROI to one half of the polygon for the tongue-to-cheek push exercise that is only performed on one side. As we aimed to reconstruct the local curvature of the cheek during the test involving (3) and (4) that can lead to cheek deformation, we used a depth camera and computed the depth map to assess the contour of the deformation in the ROI. When it came to the depth map, our first approach was to use a depth camera that could directly reconstruct the local curvature of the surface seen. The depth camera Intel Realsense D435 (Intel) has, according to the vendor, a relative accuracy below 2% for a distance less than 2 meters. This technology uses infrared and stereo cameras to analyze the deformation of a projected pattern of a scene and reconstruct from this information the depth, but requires camera calibration [25-28]. All tests were performed in realistic conditions for telehealth, that is, the distance of the face from the camera was 1 meter at minimum and the patient was directly facing the camera.

The second approach we used was to assess a pure computer vision technique that works on a standard video. Our objective was to define basic information regarding when the cheek deformation starts, when it ends, and how it may become weaker during the examination period. In practice, this is what the medical doctor may grade during a telehealth consultation.

The first solution exploits the local skin appearance alterations as the cheek becomes dilated [29]. We could then compute the ROI “centered” on the cheek area where we expect the deformation to be most significant and the average pixel value of the blue dimension of the RGB code. To track the ROI, we used the mouth location and external boundary of the cheek that can be recovered from the model (Figure 1A). We could then track the average value over time during the exercise, that is, before the push to its end. We show in the Results section the limitation of this method that is a priori not robust with respect to light conditions and may depend on skin color.

The second solution is based on the observation that cheek deformation impacts the mouth geometry. For example, in the cheek puff exercise, the mouth is closed and invariably the lip shape features change from those in the rest position. In the one-side tongue-to-cheek push, the upper lip is deformed. All these changes can be monitored in time easily by tracking the relative position of the points in the facial model that mark the mouth (Figure 1A).

We describe our computer vision methods based on an analysis performed with 3 different formats of videos. The first was acquired with our new telehealth platform using a high-definition camera with a patient who has a normal cheek puff response. The second was acquired on a control subject with a cell phone camera (Apple 13 system, Apple Inc), and the third was extracted from the MGNet data set. We tested the impact of diversity with White subjects, subjects with dark sun tan, and subjects who were African American. We demonstrate in the Results section which metrics appeared to provide the best assessment.

#### Voice Analysis

Our goal was to assess breathing and change in speech in patients with MG from analyzing counting to 50 and single-breath count. Dysarthria is not a simple concept and is classified in several ways [30]. Shortness of breath was easier to define but could be compromised by multiple factors. Shortness of breath and pulmonary function can be assessed from speech as appreciated by others [29,31]. Previous studies have used machine learning and artificial intelligence (AI) techniques that require large training sets, and they are not specific to any neurological disorder or specific to a voice acquisition protocol.

A good example of dysarthria detection has been published previously [32]. The rate of success of a neural network is modest, that is, about 70% when competing with standard diagnostic performance. An alternative solution is to use a fractal feature as reported previously [33]. This methodology seems to reach a greater accuracy of about 90% and does not require a training set.

Lip and jaw movements are related to dysarthria [34]. We are not aware of any systematic study that combines automatic lip motion tracking and speech digital analysis to assess breathing and dysarthria in patients with MG. We assessed more than half a dozen algorithms producing various sound metrics to check for the potential best voice analysis candidate to assess MG patients. As the analysis of the pitch of voice did not show any outliers in the data set and the energy metric analysis was impacted by the environment and control of the exercise, we restricted the description to the most promising algorithm. To compute voice features, we used the following steps. We separated the interval of time when the subject spoke from when the subject was silent. We used the MATLAB function “detectSpeech” [35] on the original signal. The function “detectSpeech” provides the start and end times of each so called “speech segment.” The frequency of signal acquisition was about 1000 Hz. For comparison, we used our own custom-made algorithm to extract speech segments using sampling of size 60 of the voice signal. The signal now had an equivalent frequency of acquisition of about 17 Hz. We then used averaging on each sample of the original signal to dampen noise. The signal was then smoother, and we could use a threshold to filter out noise without building up a large number of small gaps corresponding to “no sound.” We looked in the sound track of “counting to 50” exercises for the largest 50 time intervals of sound above noise level. All voice features presented below were computed on the sound track that contained speech only.

We present below the list of voice features we computed systematically for each of the sound tracks for both voice exercises. All these individual metrics or combinations of metrics were candidates to grade the severity of symptoms. The Results section reports which metric worked the best. The features are as follows:

- Loudness of voice: Loudness was computed based on the algorithms defined in the ITU-R BS.1770-4 and EBU R 128 standards. The loudness of voice was integrated over all speech segments.
- Pitch or fundamental frequency of voice: The pitch was computed for each speech segment. The speech of a typical adult man will have a fundamental frequency from 85 Hz to 155 Hz and that of a typical adult woman will have a fundamental frequency from 165 Hz to 255 Hz.
- Spectral energy on a frequency interval: Both voice exercises were considered as breathing exercises, so we computed the L2 norm spectral energy of the voice signal over all speech segments in a frequency window that focused on the breathing rate (5 Hz to 25 Hz).
- Teager-Kaiser energy: It was used in tone detection [36].
- Spectral entropy of the voice signal: Spectral entropy is a measure of spectral power distribution. Spectral entropy’s concept is based on Shannon entropy or information entropy. Spectral entropy treats the signal’s normalized power distribution in the frequency domain as a probability distribution and calculates the Shannon entropy of it. The Shannon entropy has been used for feature extraction in fault detection and diagnosis [37,38]. Spectral entropy has also been widely used as a feature in speech recognition [39] and biomedical signal processing [40].
- Special feature of the single-breath count: The airflow volume expansion during speech is in first approximation related to the square of the amplitude of the sound wave [41]. We computed the integral of the square of the amplitude of the sound wave during the time window of the patient’s speech. Since there is no calibration of the microphone, the metric might be biased. There was considerable variability of diction during this exercise. Some subjects counted more slowly, while others appeared anxious and pronounced words quickly. We computed as an additional feature the percentage of time with vocal sound versus total time.

For the voice analysis test in particular and for tests in general, there was significant variability in the parameters of data acquisition under clinical conditions, such as sound level. Providing guidance in real time to the patient will be essential to improve the ability to quantitate the telehealth examination.

### The Need for a Novel Telehealth Platform to Support the Protocol and Improvement of Data Acquisition

**Figure 3:**
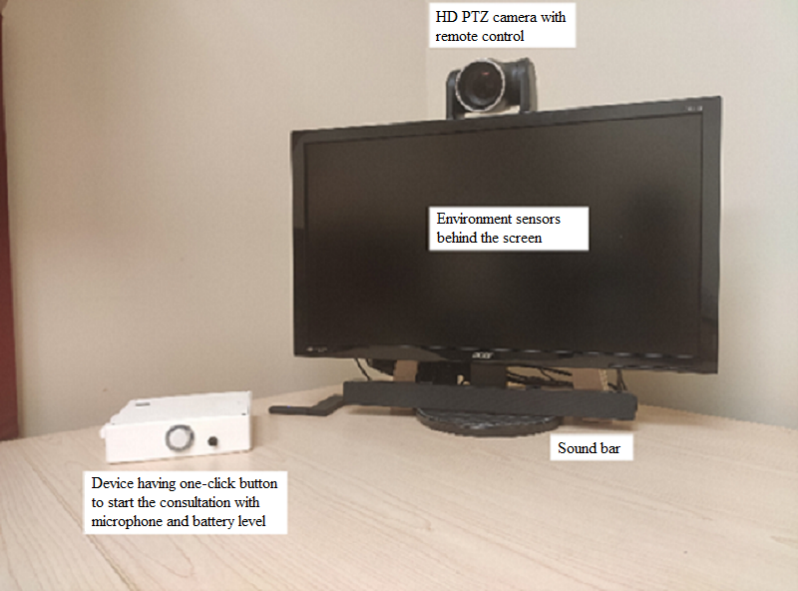
View on the patient side of our cyber-physical system named “Inteleclinic” used to uniformize the sessions and improve the quality of the metrics. HD: high-definition.

Reproducibility requires that the various examinations are run in similar conditions. While we have mainly evaluated our algorithms on an existing data set of standard Zoom video evaluations with 6 patients, we next describe our new hardware and software solution named “Inteleclinic” (Figure 3) designed to improve data acquisition.

#### Controlling the Setting and Hardware

To avoid changes in the quality of the recording (frames and audio), it is important that the hardware used is identical for all sessions and is calibrated. In an attempt to improve the quality of data acquisition with the future development of clinical studies, we built a new telehealth system with a high-definition camera and microphone that can be controlled remotely by the examiner. The recording is now performed on the patient side to obtain the raw footage of the video and audio in order to optimize maximum resolution and avoid any issues with network connection quality during the examination. Different interfaces and tools are added compared to Zoom’s control system to assist the patient and the doctor to focus on the consultation and not the technology. We demonstrate in the Results section the benefits of Inteleclinic compared to a standard Zoom video call of a patient at home using various technologies.

#### Controlling Time

Some of the tests, such as lid and eye positions as well as arm position, require precise timing from start to end. To avoid any manual entry errors, our digital heath system automatically computes start and end times. The single-breath test as practiced now has no control on loudness and timing. Breath capacity is the product of the air outflow flux and the duration of the exercise. To be more precise, breath capacity is the time integration of the time-dependent output flow on the time interval of the exercise. We found that the maximum number counted is weakly correlated with the duration of the counting exercise. The maximum number reached is dependent on diction and may not be used as a valuable metric. Outflow during speech is proportional in the first approximation to the square of the energy source of sound [41]. Depending on how loud the count is, one may expect different airflow output values for the patient. We tested on our platform a visual aid on the telehealth display to guide patient counting with a consistent rhythm of about 1 number counted per second in both the count to 50 and single-breath counting exercises.

#### Controlling the Framing of the ROI and the Distance From the ROI to the Camera

The digitalization of the tests involving ptosis, diplopia, cheek puff, tongue to cheek, arm strength, and sit-to-stand movement depends heavily on vision through the telehealth system. While we used a standard Zoom video that was preregistered in this study, it is straightforward with our system to provide guidance on the quality of video acquisition to make sure that distance between landmarks of interest use approximately the same number of pixels in order to provide quality and consistency for the results. In practice, we can provide a mark on the display of the patient with Inteleclinic to make sure the individual is properly centered and distanced from the camera.

#### Controlling Sounds

Every telehealth session may have different loudness of the sound track depending on the microphone setting and how loud or how soft the patient is speaking. Loudness is computed based on the algorithms defined in the ITU-R BS.1770-4 and EBU R 128 standards. If the microphone is calibrated with a benchmark sound, the loudness of the sound track can be computed continuously and guidance can be provided to the patient on how loud or soft they should try to keep their voice during the exercises.

### Ethics Approval

All participants provided written consent for inclusion in the study. The study that provided the data has been approved by the George Washington University Institutional Review Board (IRB# NCR224008).

## Results

### Standardization of the Data Acquisition

We evaluated our methods and identified large variability in the data acquisition and mode of operation for the assessments performed by the single examiner. The purpose of the primary study from which we obtained the videos was to assess test/retest variability and interexaminer variation in performance of telemedicine evaluations (manuscript in preparation). Our goal was to assess accurate and robust measurements from the MG-CE in order to remove human bias. The videos from the clinical study are quickly showing some limitations as the hardware used to film was not identical and the recording was performed on the doctor side, linking the quality of the frames to the quality of the network on both sides. We will next present our methodology and the platform. One of the purposes of our project was to standardize the data acquisition during the telehealth session and provide real-time feedback of the quality of the assessments for the examiner.

### Eyelid Position and Eye Movements

**Figure 4:**
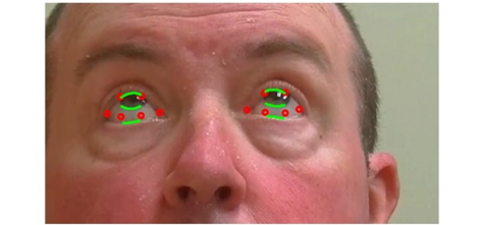
Image during the ptosis exercise. Digital zoom on the view of the patient obtained with the Inteleclinic system showing anatomic markers obtained by computer vision in green, starting from the landmarks of the regions of interest obtained by deep learning.

We have used a data set of images of 6 patients and 3 healthy subjects with broad diversity in skin color, eye color, ocular anatomy, and image frame resolution to test the accuracy of our approach. We identified 72 ROIs of patients’ eye movements and then annotated them with ground true measures obtained by manually zooming in the computer images. On average, we found the pupil location within 3 pixels and the distance from the pupil to the upper eyelid within 2 pixels independent of the image frame resolution. The relative accuracy in pixels was independent of the camera used. The standard Zoom video has 450×800 pixels per frame, a smartphone has 720×1280 pixels, and the Lumens B30U PTZ camera (Lumens Digital Optics Inc) has a resolution of 1080×1920. Overall, Inteleclinic doubles the resolution of a standard Zoom call and provides a submillimeter accuracy of lid position and eye motion. Figure 4 provides an example of the localization of the upper lid, lower lid, and iris lower boundary detected automatically with our hybrid method using digital zooming of the face of the patient with both ROIs. We underline that the patient is sitting about 1 meter from the camera during the telehealth eye exercises, and one can see the patient’s face and shoulders. No particular effort was made to focus on the eyes of the patient in the video. The 6 red circles in each eye correspond to the markers of the ROI obtained with the deep learning library of model 1 (Figure 1A). The bottom markers are slightly off, and our local computer vision technique provides the ability to correct the position of the lower lid.

**Figure 5:**
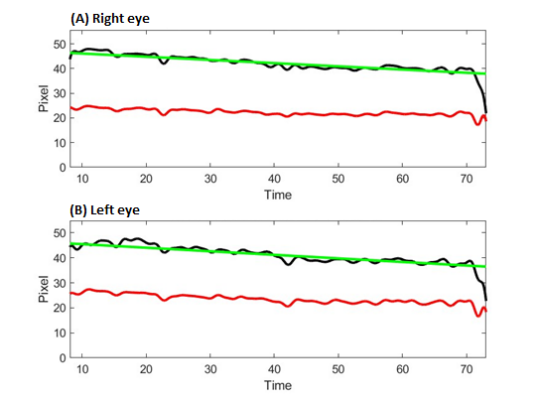
Graphic representation of the distance between anatomic landmarks to asses ptosis dynamically during the first eye exercise.

In Figure 5, we show tracking of the distance between the lower boundary of the iris and the upper lid with a black curve, and the distance between the bottom of the iris and the lower lid with a red curve. One can check that the patient performs the exercise properly and can measure a 15% decay of the ptosis distance during the 1-minute exercise. As shown in the green least square fit with the green line, this decay is both linear and statistically significant. This decay is in fact difficult to notice during a medical examination without our method.

**Figure 6:**
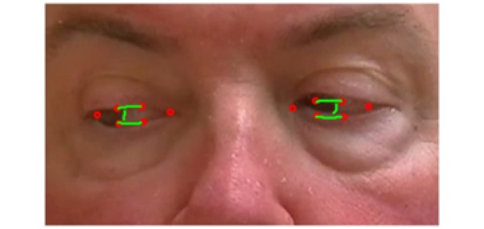
Image during the diplopia exercise. Digital zoom on the view of the patient obtained with the Inteleclinic system showing anatomic markers obtained by computer vision in green, starting from the landmarks of the regions of interest obtained by deep learning.

**Figure 7:**
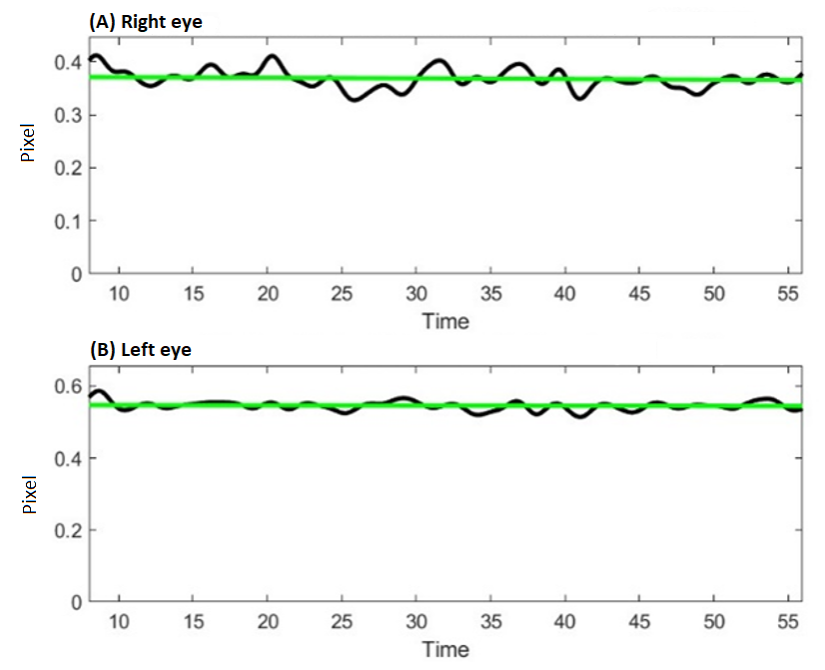
Graphic representation of the bariatric coordinate of the anatomic landmarks used to assess eye alignment dynamically during the third eye exercise.

In Figure 6, we report on the second exercise that tests diplopia. The red circle locations of the deep learning model of Figure 1A in the ROIs are accurate. We tracked the vertical border of the iris and computed the barycentric coordinate of the most inner points of the boundaries to compute any eventual misalignment of both eyes. The patient did not report double vision, and the quasisteady variation of the barycentric coordinates, as reported in Figure 7, confirmed this.

However, the positions of the eyes of patients might be so extreme that some of the pupils might be partially obstructed during the exercise, which limits the value of the conclusion. In addition, we clearly observed ptosis during the exercise as the vertical dimension of the eye opening reached about half of what it was during the ptosis exercise.

### Cheek Puff and Tongue to Cheek

We used a low-cost depth camera from Intel to reconstruct the local curvature of the cheek in laboratory conditions with a healthy subject who produced a large deformation, which was at the noise level of the signal. This evaluation would have failed for any patient who has difficulty to push the tongue into the cheek. Better depth accuracy could be obtained by sensors that use time-of-flight technology [42].

The variety of videos demonstrates the limits and potential of our approach. In one video of the cheek puff exercise, the patient was told to blow his cheeks for about 2 seconds. The video was cut after 15 seconds because the patient was asked to test the stiffness of the skin with his fingers. The placement of the fingers on the cheek completely confused the AI tracking algorithm. The change in the mean value of the third component (blue) of the RGB classification inside the ROI on both sides of the cheek of the patient is not reliable unless the left cheek or right cheek ROI has good illumination. The detection is usually far less reliable on one of these ROIs because it is difficult to achieve good illumination on both sides of the face of the patient.

**Figure 8:**
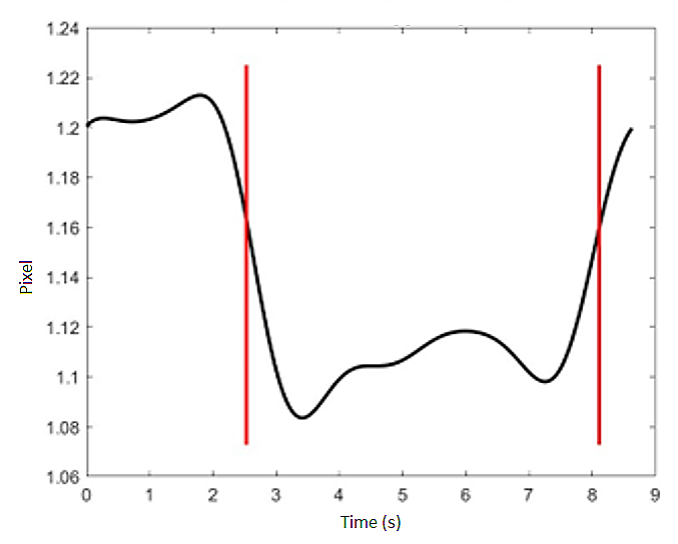
Normalized distance of the upper lip to the lower part of the nose during the cheek puff exercise.

Tracking mouth deformation during the exercise was a superior approach. First, we easily detected if and when the patient had the ability or did not have the ability to keep the mouth closed. Second, we tested several features, such as the distance between the corners where the upper and lower lip meet, that is, the segment delimited by the points (49) and (55) in the model (Figure 1A), the deformation of the mouth in the vertical direction, and the mean curvature of the upper lip and lower lip. Figure 8 shows the feature that measures the normalized distance between the upper lip and the bottom of the nose during the cheek puff exercise using a standard Zoom video with 450×800 pixels per frame in an ADAPT (Adapting Disease Specific Outcome Measures Pilot Trial) patient. We obtained a curve that was close to the step function in this ADAPT patient, which accurately detected when the deformation of the cheek started and ended, and indicated how strong the deformation was. Not all features work all the time for all patients. As expected, variability in the anatomy of patients causes differences in which features work the best. Form our experience, we found that the combination of several features helps identify the extent of cheek puff during the exercise.

**Figure 9:**
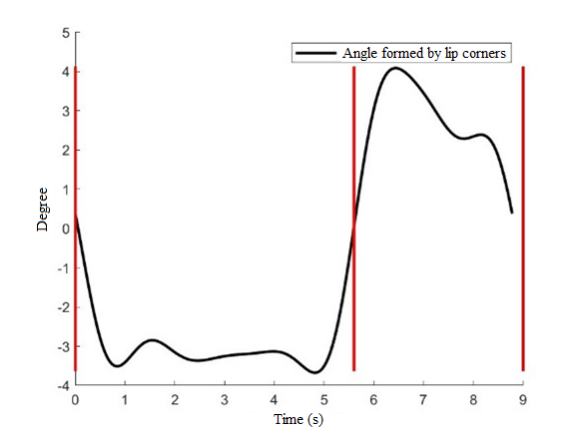
Exercise involving the tongue pushing the left cheek and then the right cheek with an ADAPT (Adapting Disease Specific Outcome Measures Pilot Trial) patient and a standard Zoom video. Tracking modifications of the lip shape orientation during the exercise. The red vertical bar in the middle corresponds to the patient switching from pushing the left cheek to pushing the right cheek.

We obtained very similar results for the tongue-to-cheek push exercise. In Figure 9, an ADAPT patient pushes the left cheek with the tongue and then pushes the right cheek at 5.6 seconds.

The geometric feature we used was the angle formed by the mouth and the horizontal axis. The exercise breaks the symmetry of the face, and this feature is particularly adapted to capture the one-side deformation of the cheek. The illumination figure shows only marginal change for the second part of the exercise and is therefore not very robust. One may expect however that better control of the light during the telehealth session will resolve this issue.

These techniques will not work for a subject with a moustache or beard. The shape of the face of patients with a high BMI may also impact the quality of the results. More work needs to be done on the digitalization of this specific test. As mentioned before, the depth camera would need to be highly accurate in order for the signal to be above the noise level, which is not the case with entry-level and low-cost systems.

### Arm Position and Sit-to-Stand Movement

Most videos of the MGNet data set offered only partial views of the body during these exercises and showed great variability. The model (Figure 1) failed under such conditions.

**Figure 10:**
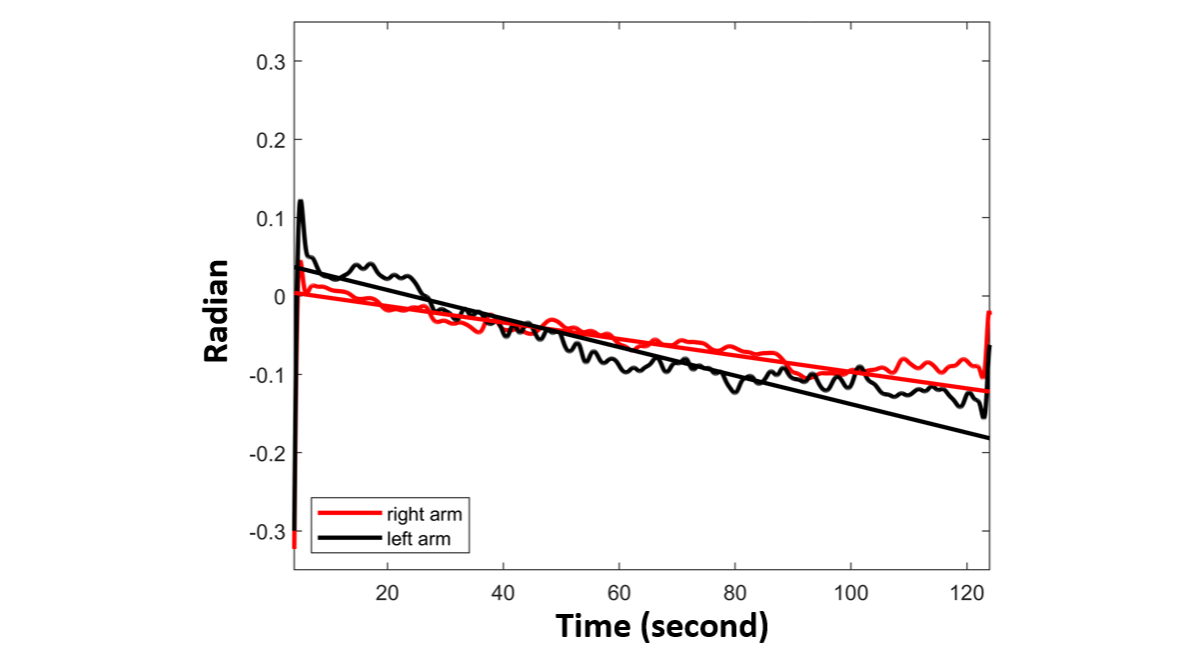
Patient performing one of the exercises of the protocol with movement of the arms. Tracking the angle of right and left arm lowering during the exercise.

Figure 10 shows a representative example of the arm angle decay due to weakening during the 120-second assessment of one of the ADAPT patients. The measurement exhibited some minor noise. We used a high-order filtering method [43] to provide a meaningful graphic to limit the noise of the method and maintain the trend that could be used for the physical examination assessment. The decay of both arms was linear and significant. It was however difficult for the medical examiner to quantify the slope or even notice it.

**Figure 11:**
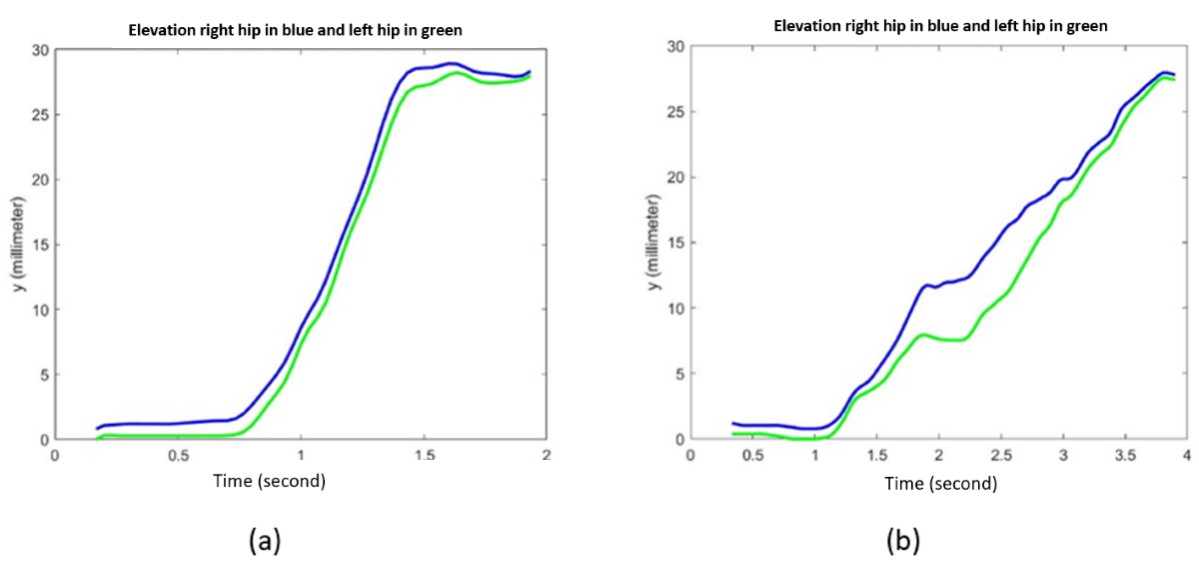
Elevation of both hips during the exercises: (A) normal stand up; (B) weak stand up. The right hip is indicated in blue, and the left hip is indicated in green.

Figure 11 shows an example of the vertical elevation examination with respect to time for both hips, involving tracking the elevation of landmark points (23) and (24) (Figure 1B) as a function of time. From this measurement, we could not only compute acceleration and speed as indicators of muscle function but also assess the stability of the motion by measuring lateral motion in the x-coordinate.

One of the benefits of having the whole body tracked during these MG-CE evaluations is the ability to access additional information, such as the ability of the patient to stay stationary and keep their balance. While all measures are in pixels in the video itself, we recovered a good approximation of the physical dimension using the known dimension of the seat.

### Counting Exercise

This evaluation is used to assess breathing and speech quality. We used a data set of 6 patients involving 2 sessions and 9 additional healthy subject voice exercises. Audio files were cut to start and end approximately within 1 to 3 seconds of initiation and the end of counting. Based on the evaluation of patients performed by the physician according to the protocol [7], there were rarely differences between the first visit and the second controlled visit for ptosis and diplopia grading. We found however that most of the metrics described above had some variability from one visit to another, and might be considered as more sensitive metrics than the current physician examination. We will report here on our main findings with these metrics.

**Figure 12:**
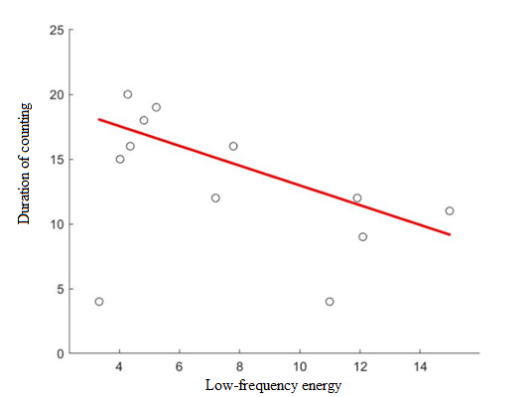
Weak correlation between the duration of counting and the energy of the signal in the low-frequency bandwidth corresponding to the breathing range. Three patient sessions with voice loudness below the threshold were not counted in the fitting. The 2 outliers are from 1 patient who had a very weak voice acquisition.

Instead of using the maximum number reached during the single-breath counting exercise, we used the duration of the exercise itself to grade the exercise. We found that the maximum number counted was weakly correlated with the duration of the single-breath counting exercise. The maximum number reached was indeed dependent on the speed of diction that varies greatly from one patient to another. To be more precise, one may expect that the airflow output value for the patient depends on the loudness of the voice and the pitch of the voice. In fact, we found that loudness and pitch computed with our algorithm varied dramatically from one patient to another. There had been no calibration of the microphone at the patient’s home, so we used the duration of the single-breath counting exercise as an indicator of MG severity. We suspected that a lower duration of the single-breath counting exercise is associated with more severe shortness of breath symptoms. We formulated the hypothesis that breathing difficulty might be detected by analyzing the signal in a range of frequencies concentrating on the typical breathing rate window. We used a fast Fourier transform to obtain the spectrum of the voice signal during the complete duration of the counting to 50 exercise and computed the energy of the signal restricted in the low-frequency window (5 Hz to 25 Hz). We found a weak correlation between the energy and MG severity estimated as described above (Figure 12). There were 2 outliers corresponding to 1 of the 6 patients who had severe symptoms according to the examiner annotation (Figure 12). The voice of the patient was so weak in the acquisition that the breathing signal information might have been at the noise level of the method.

**Figure 13:**
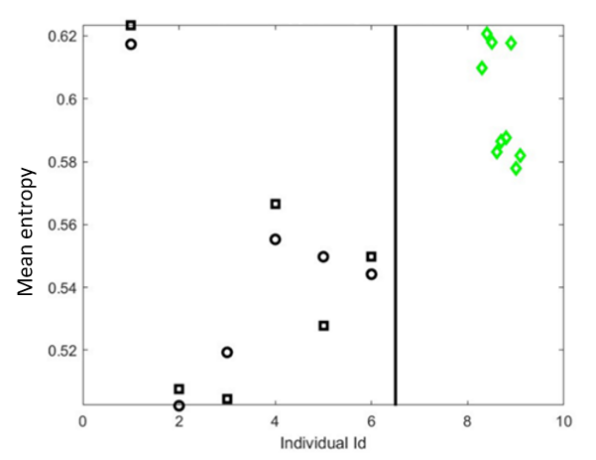
Mean Entropy of the voice signal during Exercise 7.

We did not proceed with the identification of dysarthria per say, but looked for a relationship involving one of the generic metrics that could be computed such as spectral entropy or Teager-Kaiser energy. An example of the mean entropy of the voice signal (Figure 13) shows that this criterion is promising and may separate patients from healthy controls. Counting the number of singular picks in entropy during the examination provides better separation between patients and healthy subjects. The argument would be that an MG patient has a more monotonic voice than a healthy subject. More evaluations will be needed to confirm if entropy is a good metric. In contrast, Teager-Kaiser energy did not clearly separate MG patients.

## Discussion

### Principal Findings

We have systematically built a series of algorithms that can automatically compute the metrics of the MG-CE, which is a standardized telemedicine physical examination for patients with MG. This effort was motivated by the increasing use of telemedicine and the appreciation of inherent limitations of presently used clinical outcome measures [44,45]. For the MG-CE, the examiner ranked the subjective observation of each examination item into categories, but this separation among classes was performed a priori and was not the result of data mining in a large population. In that context, the threshold numbers used to separate metrics, such as duration, are likely to be artificial. The data collection for these tests during a teleconsultation is tedious, repetitive, and demanding for the physician. We demonstrated a methodology, which can accelerate the data collection and provide the rational for a posteriori classification of MG severity based on a large population of patients.

Other ranking of the test might be intuitive, for example, how to define or compare difficulty in standing up. It may involve tedious motion due to muscle weakness, arthritis, or obesity. Currently, the duration of cheek deformation is not counted, but our methodology may eventually provide a precise measurement. Based on the new data set that our method provides, one should investigate further if the MG-CE classification, as well as all other categorical measures in MG, should be revisited to consider the new metrics that our algorithm can provide. In particular, the dynamic component of muscle weakness, which is a hallmark of MG and an important factor in quality of life, is not captured well by existing clinical outcome measures and not at all in routine clinical practice [46].

Our study exposed limitations in aspects of neuromuscular examination. The ability to deform the cheek does not say much about the ability to hold pressure and for how long. The cheek deformation exercise did prove to be the most difficult for achieving proper digitalization. The scoring of this exercise in the original medical protocol appeared particularly limited. We have refined mouth deformation monitoring under laboratory conditions with our Inteleclinic system to better apply computer learning techniques. Moreover, the counting exercises can be used to assess respiration function, but the number achieved does not fully equate to the severity of respiratory insufficiency.

Another challenge for our evaluation is that patients can compensate for some level of weakness and reduce the apparent severity assessed by the examination. For example, the ability to precisely compute the trajectory of the patient’s hip movement during the sit-to-stand exercise may identify if there is compensation by one leg supporting the movement more than the other. This situation could be particularly difficult for a human examiner to identify. Overall, our algorithms should give unbiased results and remove any potential subjectivity from the medical examination.

The accuracy of every computer algorithm must be constantly interrogated. Every metric should, in principle, come with an error estimate, which is not frequently the case in the current solutions, including that of the human examiner. One key component to ensure such quality of results is to control the condition of the acquisition of video and sound during the telemedicine session. With voice analysis, we would need to ensure proper calibration of the microphone at the patient’s home, as well as check during the sound registration that the patient speaks with a loudness within acceptable bounds. The later can be done automatically in order to provide guidance during the examination. Similarly, the AI and computer vision aspects of the data acquisition require the patient’s distance from the camera and the light condition to always be consistent with the exercise requested. This is technically feasible because the telehealth system can compute in real time the dimension in pixels of any ROI and the quality of segmentation in order to correct any obvious mistake in the data acquisition. For example, the AI model of Figure 1B that tracks the sit-to-stand exercise fails if the patient’s head leaves the video frame. This kind of problem can be immediately reported to the examiner during the test. Another example is that the single-breath counting test may poorly define the initial state, speed, and loudness of speech, as counting greatly varies between patients and has an impact on breath performance evaluation.

### Conclusion

Systematic digitalization and control of quality of the MG-CE are advantageous and would allow trained medical assistants to perform standardized examinations, allowing the physician to concentrate on patient questions and education instead of managing the logistics of the test. We also assessed our hardware-software “Inteleclinic” solution for telehealth consultation, which appears to be able to enhance data quality (described in a provisional patent; number 63305420; Garbey M and Joerger G, 2020). Our methods and technology would be particularly applicable to clinical trials, which are limited in requiring a large number of examiners who all perform assessments in slightly different manners. A trial could substitute present operations with a central telemedicine facility. We envision that our telehealth approach can be applied to other neuromuscular diseases beyond MG and will provide objective, reproducible, and quantitative health care assessments that go beyond the present capabilities.

## Abbreviations

ADAPT: Adapting Disease Specific Outcome Measures Pilot Trial
AI: artificial intelligence
MG: myasthenia gravis
MG-CE: Myasthenia Gravis Core Examination
ROI: region of interest
